# Effect of Cereal α-Amylase/Trypsin Inhibitors on Developmental Characteristics and Abundance of Digestive Enzymes of Mealworm Larvae (*Tenebrio molitor* L.)

**DOI:** 10.3390/insects12050454

**Published:** 2021-05-14

**Authors:** Sorel Tchewonpi Sagu, Eva Landgräber, Ina M. Henkel, Gerd Huschek, Thomas Homann, Sara Bußler, Oliver K. Schlüter, Harshadrai Rawel

**Affiliations:** 1 Institute of Nutritional Science, University of Potsdam, Arthur-Scheunert-Allee 114-116, 14558 Nuthetal, Germany; sorelsagu@uni-potsdam.de (S.T.S.); e.landgraeber@live.de (E.L.); homann@uni-potsdam.de (T.H.); 2 EntoNative GmbH, Arthur-Scheunert-Allee 40-41, 14558 Nuthetal, Germany; ina.henkel@tenetrio.de; 3 IGV-Institut für Getreideverarbeitung GmbH, Arthur-Scheunert-Allee 40-41, 14558 Nuthetal, Germany; gerd.huschek@igv-gmbh.de; 4 Leibniz Institute for Agricultural Engineering and Bioeconomy (ATB), Quality and Safety of Food and Feed, Max-Eyth-Allee 100, 14469 Potsdam, Germany; sbussler@gnt-group.com (S.B.); or oliver.schluter@unibo.it (O.K.S.); 5 Department of Agricultural and Food Sciences, University of Bologna, p.zza Goidanich 60, 47521 Cesena, Italy

**Keywords:** *Tenebrio molitor* larvae, growth behavior, feeding, cereal meals, α-amylase/trypsin inhibitors, digestive enzymes quantification, LC-MS/MS

## Abstract

**Simple Summary:**

The main nutritionally relevant proteins of *Tenebrio molitor* L. larvae are cereal proteins. Cereals contain α-amylase/trypsin inhibitors (ATIs) that interact with digestive enzymes and which may impair the growth of the larvae. Despite the existing work on the subject, there is still a lack of information regarding the effects of ATIs on the relative abundance of various enzymes in larvae. Our aim was therefore to undertake an assessment of the potential effects of ATIs on the growth parameters and digestive enzyme contents of *T. molitor*. The larvae were fed with cereal meals containing different levels of ATIs. The developmental characteristics were evaluated and finally an analytical method based on liquid chromatography with tandem mass spectrometry (LC-MS/MS) was developed to quantify the relative abundance of enzymes in the larvae. The results indicated an increase in pupation and significantly higher protein concentrations in larvae fed with wheat meals compared to those fed with sorghum meals. Patterns of specific α-amylase activity (in mM maltose/min/mg protein) were similar to those of amylase activity (in mM maltose/min) and the larvae fed on high-ATI-content meals exhibited an increased death rate, although the results were not always significantly consistent. The results of the LC-MS/MS analysis showed a decrease of about half of the relative content of α-amylase among the three proteases monitored, as well as an increase in dipeptidylpeptidase I and chymotrypsin, whereas trypsin remained constant. Therefore, these results indicate that meal composition has an effect on the expression of *T. molitor* digestive enzymes.

**Abstract:**

The objective of this work was to investigate the potential effect of cereal α-amylase/trypsin inhibitors (ATIs) on growth parameters and selective digestive enzymes of *Tenebrio molitor* L. larvae. The approach consisted of feeding the larvae with wheat, sorghum and rice meals containing different levels and composition of α-amylase/trypsin inhibitors. The developmental and biochemical characteristics of the larvae were assessed over feeding periods of 5 h, 5 days and 10 days, and the relative abundance of α-amylase and selected proteases in larvae were determined using liquid chromatography tandem mass spectrometry. Overall, weight gains ranged from 21% to 42% after five days of feeding. The larval death rate significantly increased in all groups after 10 days of feeding (*p* < 0.05), whereas the pupation rate was about 25% among larvae fed with rice (*Oryza sativa* L.) and *Siyazan*/*Esperya* wheat meals, and only 8% and 14% among those fed with *Damougari* and *S35* sorghum meals. As determined using the Lowry method, the protein contents of the sodium phosphate extracts ranged from 7.80 ± 0.09 to 9.42 ± 0.19 mg/mL and those of the ammonium bicarbonate/urea reached 19.78 ± 0.16 to 37.47 ± 1.38 mg/mL. The total protein contents of the larvae according to the Kjeldahl method ranged from 44.0 and 49.9 g/100 g. The relative abundance of α-amylase, CLIP domain-containing serine protease, modular serine protease zymogen and C1 family cathepsin significantly decreased in the larvae, whereas dipeptidylpeptidase I and chymotrypsin increased within the first hours after feeding (*p* < 0.05). Trypsin content was found to be constant independently of time or feed material. Finally, based on the results we obtained, it was difficult to substantively draw conclusions on the likely effects of meal ATI composition on larval developmental characteristics, but their effects on the digestive enzyme expression remain relevant.

## 1. Introduction

The yellow mealworm, *Tenebrio molitor* L. (Coleoptera: Tenebrionidae), is a species of beetle insects belonging to the group of stored grain-related pests that use grains and their products as a primary food source [1]. The development of the beetle occurs through several stages, including the laying of about 400 to 500 eggs on average, the development of the larvae, which change from a whitish to a yellowish brown color and can reach 2.5 cm in length, with pupal stages that can be up to eight months under favorable growth conditions, and finally the eventual development into beetles, which can survive for about 96 days [2,3].

*T. molitor* larvae contain about 56% water, 18% protein and 22% fat (fresh weight), whereas in the dried weight, the protein and fat contents are about twice as high [4]. More recently, studies showed that the proximal composition of *T. molitor* consists of 55.6–69.8% moisture, 8.9–21.9% fat, 13.7–27.6% crude protein, 0.9–1.5% ash, and 3.1–4.8% of other components, whereas on a dry weight basis, they consist of 34.5–38.3%, 45.6–49.1%, 4.1–4.8% and 8.5–16.0% fat, protein, ash and other components, respectively [5,6]. The fats are mainly monounsaturated fatty acids (41.0%), polyunsaturated fatty acids (35.1%) and saturated fatty acids (23.0%) [5]. Mealworm larvae also contain macroelements such as calcium (0.21%), phosphorus (1.06%), sodium (0.21%), potassium (1.12%), magnesium (0.30%), as well as microelements such as ferrum (71.5 mg/kg), zinc (138.2 mg/kg), copper (19.4 mg/kg) and manganese (5.7 mg/kg) [7].

The main nutritionally relevant proteins of *T. molitor* are cereal proteins. The major fraction of the storage proteins of cereals are prolamins, which constitute as much as 50% of the total seed protein. Prolamins contain 30–50% glutamine and 10–30% proline residues [8,9,10]. Therefore, it has been suggested that *T. molitor* has digestive enzymes that can specifically and efficiently cleave peptide bonds containing these amino acid residues [11]. A complex enzymatic system of protein digestion was found within the gut of the larvae. The diversity of digestive peptidases in the gut of *T. molitor* larvae is derived from the insect itself or the microbiome present in the gut [12,13]. For tenebrionids, protein digestion is a compartmentalized process that relies heavily on cysteine peptidases due to a pH gradient that regulates the enzyme activity [14]. Diverse sets of peptidases are localized in different areas of the gut, depending on this sharp pH gradient [12,13]. The pH gradient ranges from 5.6 in the anterior midgut to 7.9 in the posterior midgut, resulting in restricted activity of digestive enzymes in different areas of the larval midgut [12]. Cysteine peptidases and carbohydrases are located mainly in the anterior midgut due to their acidic pH optima, and serine peptidases are found mainly in the posterior midgut due to their neutral or alkaline pH optima [11,12,15,16]. Among the most important protein-cleaving enzymes in the digestive tract are trypsin [17]. Trypsin, along with chymotrypsins, belong to the superfamily of serine endoproteinases [18]. *T. molitor* larvae have at least four trypsin-like and five chymotrypsin-like serine peptidases for protein digestion [12,19,20].

α-amylase is an important larvae enzyme in carbohydrate metabolism and carbohydrates, used as the main energy source for metabolic processes, and is an indispensable diet component for its survival [21,22]. *T. molitor* larvae contain a single α-amylase that is an acidic protein with a pH optimum for the cleavage of starch of 5.8, and this enzyme is accordingly well adapted to its physiological environment in the larval midgut, where a slightly acidic pH is prevalent [23]. The mature enzyme consists of 471 amino acids with a molecular weight of 51.3 kDa and a calculated pI of 4.3 (https://www.uniprot.org, accessed on 16 April 2021). The primary structure of *T. molitor* α-amylase is more than 57% identical to the known α-amylase sequences from insects and exhibits the same characteristic differences compared to mammalian enzymes as the other insect α-amylases [24,25].

The ingestion of an enzyme inhibitor provokes the overexpression of existing or ‘de novo’ expression of new digestive enzyme isozymes that are insensitive to inhibitors, whereas sensitive isozymes can be either up- or downregulated [26]. In this context, the production of non-functional digestive enzymes which serve as bait for inhibitors, the induction of specific inhibitor-insensitive enzymes, the increased consumption of inhibitor-containing food and the involvement of symbiotic microorganisms in protein digestion have also been reported [26,27]. The occurrence of multiple digestive enzyme isoforms may provide adaptive advantages for insects feeding on plants containing inhibitors [27]. Insects are usually adapted to inhibitors from their host plants, whereas specific compensatory responses of proteinases to non-host plant inhibitors are lacking or are insufficient to prevent various adverse effects on insect feeding, growth and fitness traits.

Many digestive enzyme inhibitors originating from plants are protein molecules, including the group of cereal α-amylase-trypsin (protease) inhibitors (ATIs) [28,29]. In fact, ATIs are bifunctional proteins, having the ability to inhibit both amylase and protease activities [30]. They are relatively small proteins with molecular weights ranging from 10 to 16 kDa, containing 124–168 amino acids with 10 cysteine residues, leading to the development of intramolecular disulfide bonds [31]. The abundance of ATIs in cereals depends on the species and cultivar. In the online protein database UniprotKB, 13 reviewed wheat ATIs were registered as of 7 April 2021, whereas *sorghum bicolor* L. and rice *Oryza sativa L.* (poales: Poaceae) contained two and nine different ATIs, respectively (https://www.uniprot.org, accessed on 16 April 2021). ATIs are natural components of cereal proteins, making cereals more resistant to insect attacks and thus providing a protective function in cereals, having a stronger effect on insect α-amylases and negatively affecting insect fitness, digestion and development processes [32].

Many studies have been conducted in order to investigate the interaction between cereal ATIs and the α-amylase/proteases in *T. molitor* larvae. Cuccioloni et al. [33] characterized the kinetics, equilibrium parameters and binding modes of the complexes formed between wheat *Triticum aestivum* L. (Poales: Poaceae) ATIs and *T. molitor* trypsin and alpha-amylase. Compensatory mechanisms of adaptation to enzyme inhibitors have been described for the black beetle family [34,35,36]. Feng et al. [37] investigated the patterns of inhibition of insect and human α-amylases by the α-amylase inhibitors from wheat. The interaction of wheat monomeric and dimeric protein inhibitors with alpha-amylase from yellow mealworm has also been analyzed [38]. In a recent study, 54 wheat genotypes were analyzed for inhibitor activity in vitro against *T. molitor* α-amylase and trypsin [29]. Despite the existing work on the subject and the available data, there is still a lack of experimental studies evaluating the effects of these cereal inhibitors on the expression and relative abundance of *T. molitor* digestive enzymes, and this remains a subject of interest.

The aim of the present work was to attempt an assessment of the potential effect of cereal ATIs on the growth parameters and digestive enzyme contents of *T. molitor* larvae. Different groups of *T. molitor* larvae were fed with wheat, sorghum and rice flours containing distinct levels of amylase/protease inhibitors. Developmental characteristics such as weight, death rate, live pupae and intake feed were evaluated after 5 h, 5 days and 10 days of feeding, and finally a liquid chromatography with tandem mass spectrometry (LC-MS/MS)-based method was developed to quantify the relative abundance of the larvae α-amylase and proteases.

## 2. Materials and Methods

### 2.1. Biological Materials and Chemicals

*T. molitor* larvae, approximately 4 weeks old at the beginning of the experiment, were provided by EntoNative GmbH, Nuthetal, Germany. *Siyazan* and *Esperya* wheat cultivars of the genus *Triticum aestivum* L. (from Central Anatolia Turkey) were provided by the University of Altınbaş, Turkey. *Damougari* and *S35* sorghum cultivars (*Sorghum bicolor* L. Moench) were obtained from the Institute of Research and Agronomic Development, IRAD, Maroua, Cameroon. Both sorghum and wheat samples were selected on the basis of their ATI content, determined by mass spectrometry according to the methods reported in our previous works [30,39]. Wheat *Siyazan* and sorghum *Damougari* contained low amounts of ATIs, whereas wheat *Esperya* and sorghum *S35* samples had high ATI contents. In addition, whole rice flour (Item Nr. 2618, Bauckhof, Bauck GmbH, Rosche, Germany) was used as the third type of cereal in order to better assess the feeding process results. Table 1 presents the main characteristics of the selected samples.

Trypsin of proteomics grade (M150-1G, Sigma-Aldrich Chemie, Steinhagen, Germany) was used for the in-solution digestion. In order to standardize the data from the tandem mass spectrometry analysis, a synthetic peptide consisting of four amino acids (GWGG) with a mass of 376.2 Da (Bachem AG, Bubendorf, Switzerland) was used as internal standard (IS). Solvents used for separations were of HPLC grade and all other chemicals involved in this work were of analytical grade.

### 2.2. Design and Implementation of the Feeding Experiments

**Figure 1:**
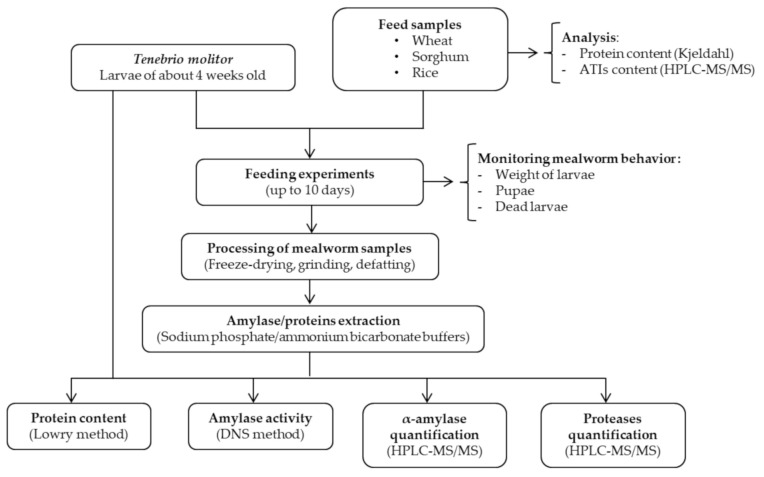
Workflow presenting the different experimental steps implemented in this study, as well as the analysis performed.

Figure 1 outlines the steps of the procedure implemented in this study. Feed samples were first characterized in terms of their protein and ATI content. The feeding experiments were conducted for up to 10 days and different analyses were performed.

#### 2.2.1. Feeding Experiments

For each experimental cycle, a quantity of 20 g each of wheat, sorghum and rice flour was placed into the small containers (17 × 10 × 8 cm). Subsequently, 10 g of larvae were introduced in the containers (ratio flour/larvae of 2:1). The average weight of larvae at the beginning of the experiment was about 0.11 ± 0.01 g (mean of the weights per larva of all groups). The total number of larvae per group was 90 ± 2. The containers were then placed in a dark room with a temperature of 25 °C ± 2 °C and 50–60% RH throughout the study period. Figure S1 (see Supplementary Data) presents an example of the containers with cereal flours and larvae. During the entire feeding period, the flours were neither replaced nor replenished. In addition to the flours, the larvae received about 5 g of fresh carrot slices daily. The weight, mortality and pupation of the larvae were documented after 5 h, 5 days and 10 days of feeding in order to follow their development process. A detailed summary of the experimental times observed is provided in Figure S2 (Supplementary Data). All the experiments were randomized and performed in triplicate. A group of larvae without intervention was collected on the first day and frozen at −20 °C. Likewise, at the end of the experiments, the larvae were collected and stored at −20 °C, and the remaining amounts of cereal flours in the containers were also weighed. The larvae were removed from the freezer and were subsequently rough-crushed before being freeze-dried. Finally, the larvae were ground with an electric grinder and the powder stored at −20 °C for further analysis.

#### 2.2.2. Sample Preparation for Amylase Activity

In order to assess the amylase activity, 200 mg of larvae powders were first defatted with n-hexane (ratio 1:6, *w/v*) and after centrifugation, the supernatant was discarded and the precipitate was air-dried under a fume hood for one hour. Subsequently, 1.6 mL of extraction buffer (2 mM sodium phosphate buffer, pH 6.9 and 300 mM, containing sodium chloride) was then added and mixed for 30 min at 4 °C. The samples were then centrifuged at 5000× *g* at 4 °C for five minutes. The clear supernatants containing amylase were collected for the determinations of amylase activity and protein content.

The amylase activity was determined using the dinitrosalicylic acid method as described by Sagu et al. [40]. Briefly, 100 μL of extract were mixed with 100 μL of 0.02 M sodium phosphate buffer, pH 6.9, containing 0.6 mM sodium chloride, and then 200 μL of 1% (*w/v*) starch solution were added. After incubation at 37 °C for ten minutes, 400 μL of dinitrosalicylic acid reagent was added. The mixtures were then incubated at 100 °C for 5 min and, after cooling at room temperature, 700 μL of distilled water was added and the absorbance was measured at a wavelength of 540 nm. The calibration curve was performed using maltose solutions at different concentrations ranging from 0.25–5 µmol/mL and the results were expressed as mM maltose equivalent per minute.

The protein content of the extracts was determined according to the Lowry method [41] using BSA as a standard, and the results were expressed as mg of protein per mL of extract. Total raw larval protein was measured using the Kjeldahl method (N × 6.25). All analyses were performed in triplicate.

### 2.3. Quantification of α-Amylase and Proteases Using Tandem Mass Spectrometry

To assess the effect of cereal ATIs on larval enzymes and consequently their impact on larval growth, an HPLC-MS/MS based analytical method was developed in order to quantify the enzymes involved at different growth stages during the 10-day feeding experiment.

#### 2.3.1. Sample Preparation for Mass Spectrometry

Prior to the digestion, 100-mM ammonium bicarbonate buffer, containing 4 M urea, was used in order to maximize the protein extraction. Briefly, 100 mg of larvae flour were first defatted in 600 µL n-hexane. The samples were then mixed for 10 min at room temperature. After centrifugation at 7000× *g* (4 °C, 10 min), the supernatant was discarded and the defatting procedure was repeated twice. Samples were dried and the defatted flours were then mixed with 1 mL of extraction buffer at room temperature under shaking conditions (95 rpm) for one hour, and were finally centrifuged (7000× *g*, 5 min, 4 °C).

Supernatants (0.4 mL) were collected and transferred to a 1.5-mL reaction tube. Ten microliters of 0.5 mg/mL β-lactoglobulin, serving as a control for tryptic digestion, was added to each sample. A blank was used, containing 0.4 mL extraction buffer and 10 μL of 0.5 mg/mL β-lactoglobulin. Ten microliters of 0.25 M tris(2-carboxyethyl)phosphine were added and the mixture was incubated at 50 °C for 20 min. Subsequently, 10 μL of 0.25 M iodoacetamide was added to the samples and incubated for another 20 min at 50 °C in the dark. Then, 135 μL of digestion buffer (100 mM ammonium bicarbonate) and 20 μL of 4 mg/mL trypsin solution were pipetted into the samples. The mixtures were incubated at 37 °C overnight under shaking conditions, and the digestion was stopped by adding 15 μL of 40% formic acid.

Peptides obtained from the digestion were first purified prior to the LC-MS/MS analysis using solid phase extraction (SPE) by applying the sample to a column constituted of octadecyl (ODS, C18 ec, RP18 ec)-modified silica phase (Chromabond C18 ec; MACHEREY-NAGEL GmbH & Co. KG, Dueren, Germany). Briefly, the columns were first activated with 6 mL of buffer A (distilled water/acetonitrile/formic acid, 50:50:0.1; *v/v/v*) and conditioned with 6 mL of distilled water. Samples were then loaded onto the columns and washed with 6 mL of distilled water. Finally, the target components were eluted from the column with 1 mL of buffer B (acetonitrile/formic acid, 100:0.1; *v/v*), filled up to 5 mL with distilled water and stored at −20 °C until mass spectrometric analysis.

#### 2.3.2. LC–ESI–MS/MS Method Development

In order to quantify the amylases and proteases of the larvae, a liquid chromatography (LC) coupled to tandem mass spectrometry (MS/MS)-based method was developed and optimized. For this purpose, yellow mealworm α-amylase (UniProtKB-P56634), as well as 6 proteases, namely modular serine protease zymogen (UniProtKB-B1B5K3), CLIP domain-containing serine protease (UniProtKB-B1B5K1), chymotrypsin 1 (UniProtKB-A1XG72), dipeptidylpeptidase I (UniProtKB-A0A286MG49), C1 family cathepsin B24 (UniProtKB-A0A0B5J553) and trypsin-like serine protease (UniProtKB-H8ZZ80), were selected for the analysis. Table S4 (Supplementary Data) presents the different characteristics and functions of the selected enzymes. The primary sequences of the protein chains of these enzymes were downloaded from the UniProtKB online database in the FASTA format (https://www.uniprot.org/, accessed on 23 October 2020), and then imported into Skyline software. The in silico digestion was subsequently performed using trypsin as the digesting enzyme, filtering out peptides with a length of 3–30 amino acids and selecting carbamidomethylation of cysteine residues as the structural modification. Precursor charge 2 and single charge fragments (*m*/*z* ratio between 50 and 1500) were considered.

Digested samples were then separated on a reversible phase Kinetex C8 column (150 mm × 4.6 mm; 2.6 µm; Phenomenex, Torrance, CA, USA); installed on an Agilent Infinity 1260 LC system consisting of a binary pump, a multi-column thermostat and a VL vial sampler. Separation was performed at a rate of 0.5 mL/min in gradient mode, using 0.1% formic acid in water as eluent A and 100% acetonitrile as eluent B. The elution program was set as follows: 0.01–2.0 min, 5% solvent B; 2–18 min, 50% solvent B; 18–19 min, 95% solvent B; 19–22 min, 95% solvent B; 22–23 min, 5% solvent B; and 23–28 min, 5% solvent B. The eluted peptides were analyzed by multiple reaction monitoring (MRM) with a dwell time of 20 ms using an Agilent G6470A Series Triple Quad HPLC/MS mass spectrometer (Agilent Technologies Sales and Services GmbH and Co.KG, Waldbronn, Germany). The collision gas used was nitrogen at a pressure of 3.85E-5 Torr and a cell gas pedal voltage of 5 kV. Electrospray ionization (ESI) was performed in positive ion mode at a desolvation temperature of 275 °C using nitrogen at a flow rate of 11.0 L/min, a capillary current of 5425 nA and nebulizer pressure of 35.0 psi.

The results were then analyzed using Skyline software. Several peptides were initially selected per analyzed protein, and after an individual optimization of the fragmentor energy, as well as the collision energy, 2 to 3 peptides were finally selected for each enzyme: one quantifier peptide (biomarker) used to perform the quantification calculations and one to two peptides serving as qualifiers for better assessment of the analysis. The biomarkers were selected on the basis of 4 criteria: the specificity of the peptide, the length of the peptide sequence, the intensity and consistency of the signal, and when possible, peptides containing cysteine residues were not considered. The specificity of the generated peptides was evaluated using both the function “Unique Peptides” in Skyline software and the BLAST function (Basic Local Alignment Search Tool) in the UniProt database. Following the establishment of the method, it was adequately validated using classical statistical tools such as linearity, selectivity, specificity, limit of detection, limit of quantification, repeatability and recovery. The effect of the matrix was also assessed. Finally, for the analysis of the samples, the digested enzymes were mixed with the internal standard (IS) peptide GWGG (ratio 1:11, *v/v*) for a final concentration of the IS of 0.1 μg/mL, and a volume of 11 μL of the mixture was injected into the HPLC-MS/MS instrument. The results were processed with Skyline software and the peak areas of the samples were normalized by taking into account the ratios between the values of the IS in water and in the sample matrix.

Following the development and optimization of the HPLC-MS/MS method, a unique peptide (biomarker) was selected for each of the analyzed enzymes in order to proceed with the quantitative measurements. The selected biomarkers were NCELVGLR, VTFTLSR, VQVPVK, TAAYR, LELAMSR, SAFIDGK and NSLLPDGR, corresponding to the enzymes α-amylase; modular serine protease zymogen; CLIP domain-containing serine protease; chymotrypsin; dipeptidylpeptidase I; C1 family cathepsin B24; and trypsin-like serine protease, respectively.

### 2.4. Statistic Analysis

All the feeding experiments, as well as the analyses, were performed and replicated three times (*n* = 3). The statistical analysis was performed using two-way ANOVA, followed by the Tukey multiple comparisons test (GraphPad Prism 6, GraphPad Software, San Diego, CA, USA). The data were presented as mean ± standard deviation and the results were considered statistically different for *p*-values less than 0.05 (95% confidence interval).

## 3. Results

### 3.1. Developmental Characteristics of Tenebrio molitor Larvae during the Feeding Experiments

**Figure 2:**
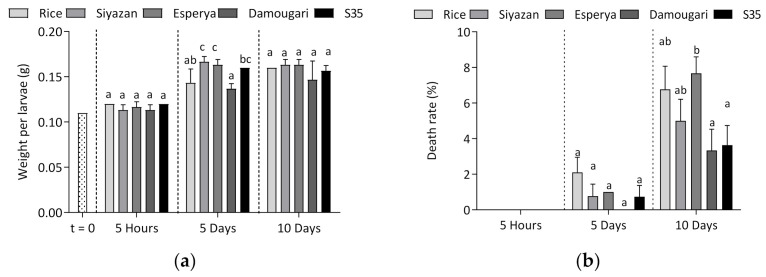

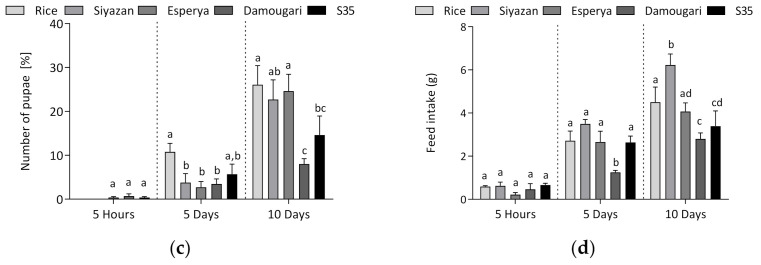
Changes in (**a**) weight of larvae, (**b**) larval death rate, (**c**) number of live pupae and (**d**) feed intake as a function of feeding duration. Different letters indicate statistically significant differences at *p* = 0.05 (Tukey test).

During the feeding experiment, the weight, death rate and percentage of pupal survival were recorded. In addition, the amount of feeding samples ingested was documented. The data are presented in Figure 2. The average weight of each larva at the beginning of the experiment was around 0.11 ± 0.01 g. They showed similar weights after 5 h of feeding within the different groups, whereas the larvae from both wheat flours (*Siyazan* and *Esperya*) showed significantly higher weights (*p* < 0.05) after 5 days compared to those from whole rice flour and sorghum flour *Damougari* (Figure 2a). Overall, weight gains ranged from 21% to 42% after five days of feeding. Larvae fed with sorghum flour *Damougari* with lower ATI content demonstrated in the lowest weight gains, whereas those fed with *Esperya* wheat flour (with a higher ATI content) exhibited the highest weight gain. The larvae within the 10-day groups showed weight averages of 0.15 to 0.16 g, similar to those of the 5-day groups. However, it was observed that larvae fed with the sorghum *Damougari* flour exhibited lower weights compared to those fed with the other samples (Figure 2a).

Figure 2b: after 5 days of feeding, the larvae from rice flour tended to show the highest death rate (2%), whereas no deaths were reported for the larvae from the sorghum sample *Damougari*, and the analysis of variance showed that the differences in these results were not significant. However, a significant increase in the death rate was observed after 10 days of feeding for larvae from all flour samples. The death rate ranged from 3% to 8%; with wheat flour *Esperya* with higher ATI content exhibiting the higher death rate, whereas the two sorghum flours *Damougari* and S35 (which contain less ATIs in comparison to rice and wheat flours) led to a lower death rate (Figure 2b). Similar trends were observed with the number of pupae. The proportion of live pupae varied between 3% and 11% after a feeding period of 5 days. After 10 days of feeding, a pupation rate of about 25% was observed in larvae fed with rice and *Siyazan* and *Esperya* wheat meal, whereas only 8% and 14% of pupae were recorded over the same period in the sorghum meal *Damougari* and S35 groups, respectively (Figure 2c). It appears that after 10 days, feeding the larvae with sorghum meal resulted in a significant reduction of the pupation, compared to wheat and rice flours.

### 3.2. Effect of Feeding Cereals on Larvae Protein Content and Amylase Activity

#### 3.2.1. Protein Content

The results regarding the protein content of the extracts are presented in Table 2. It appeared that of all the samples, the sodium phosphate extracts yielded protein concentrations in the range of 7.80 ± 0.09 to 9.42 ± 0.19 mg/mL, whereas the Ambi/urea extracts exhibited protein contents ranging from 19.78 ± 0.16 to 37.47 ± 1.38 mg/mL. From the sodium phosphate extracts, the larvae fed with wheat flours *Siyazan* and *Esperya* showed significantly higher protein concentrations (*p* < 0.05) compared to those fed with rice and sorghum flours within the 5-day and the 10-day groups. There was no difference between the larvae from the same experimental groups fed with wheat flour containing low and high amounts of ATIs (Table 2). This contrasts with the results of the Ambi/urea extracts. In Table 2 it can be observed that after 5 and 10 days of feeding, the protein levels extracted using the Ambi/urea buffer tended to be significantly higher in larvae fed with rice and sorghum meals (*p* < 0.05). In addition, an increase in protein concentrations was initially observed between days 1 and 5, followed by a clear decrease after day 10 of feeding. Finally, the total protein contents of larvae were determined according to the Kjeldahl method and the values ranged from 44.0 to 49.9 g/100 g. Overall, a decrease in the total protein value of the larvae was observed with the feeding time, regardless of the type of meal given to the larvae. Thus, as a whole, the protein values, which were about 53.4 g/100 g before the beginning of the experiments, fell to an average of 49 g/100 g after only 5 h of feeding. The decrease in the protein level persisted throughout the experiment, falling successively to an average of 46% and 45% after days 5 and 10, respectively.

#### 3.2.2. Effect of Feeding with Cereals on α-Amylase Activity

**Figure 3:**
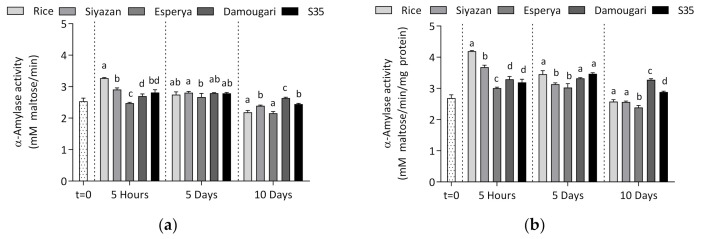
Evolution of (**a**) α-amylase activities and (**b**) specific α-amylase activities of larval extracts as a function of intake type and feeding duration. Different letters indicate statistically significant differences at *p* = 0.05 (Tukey test).

The determination of the α-amylase activity of larvae after the feeding experiment was performed using the dinitrosalicylic acid method and the results are presented in Figure 3. When comparing the 5-h group with the initial larvae just before starting the feeding experiments, it appears that there was an increase in the amylase activity in the larvae fed with rice, wheat cultivar *Siyazan* and sorghum cultivars *Damougari* and *S35* flours. The highest α-amylase activity within the 5-h group was exhibited by larvae fed with whole rice grain flour (3.27 ± 0.02 mM maltose/min). Conversely, feeding larvae with wheat (*Esperya*), with a high content of ATIs, yielded significantly lower α-amylase activity (2.47 ± 0.03 mM maltose/min). The results also suggest that feeding larvae with sorghum flours *Damougari* or *S35* did not result in significantly different α-amylase activity *p >* 0.05).

Feeding the larvae for 5 days, the α-amylase activity activities ranged from 2.67 ± 0.11 mM maltose/min (larvae fed with wheat cultivar *Esperya*) to 2.81 ± 0.04 mM maltose/min (larvae from wheat cultivar *Siyazan*) (Figure 3a). The statistical analysis revealed that there were no significant differences between the activity values. However, the α-amylase activities tended to be significantly lower after 10 days of feeding, compared to those after 5 days, and the values ranged from 2.16 ± 0.06 to 2.64 ± 0.03 mM maltose/min.

Taking into account the protein content, the specific activities of the larvae extracts were also evaluated and the results are presented in Figure 3b. It emerged that the patterns of the specific activities according to the type of feed provided to the larvae were similar to those of the amylase activities. It was also observed that within both the 5-h and 10-day groups, the extracts from that the larvae fed on the wheat cultivar with high ATI content (cultivar *Esperya*) exhibited significantly lower α-amylase activity, compared to those fed with the wheat cultivar *Siyazan,* which contained a low amount of ATIs (*p* < 0.05). In contrast, with both higher and lower ATI contents, sorghum flours showed significantly differences only for the group of larvae fed for 10 days.

### 3.3. Determination of the Relative α-Amylase Content in T. molitor

#### 3.3.1. Method Development and Validation

Table S5 (Supplementary Data) presents the final optimal parameters used to determine the relative content of mealworms α-amylase and selected proteases by means of the targeted LC-MS/MS method. Linearity was assessed (see Figure S3, Supplementary Data); the developed method was adequately validated using conventional statistical tools and the investigated parameters indicated the suitability of the method.

#### 3.3.2. Evaluation of Relative Abundance of Larval α-Amylase and Selected Proteases

**Figure 4:**
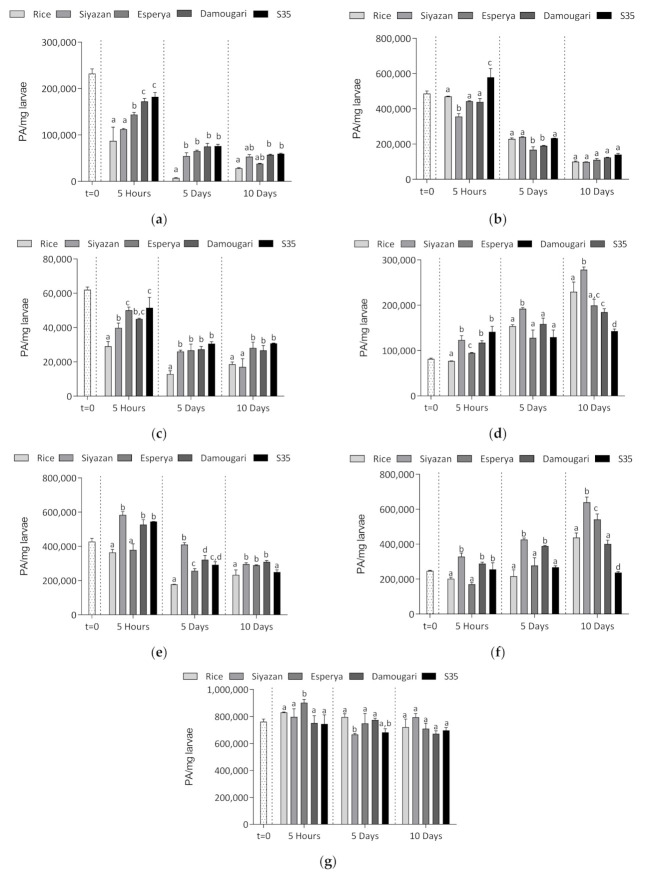
Changes in terms of relative content of (**a**) α-amylase, (**b**) CLIP domain-containing serine protease, (**c**) modular serine protease zymogen, (**d**) dipeptidylpeptidase I, (**e**) C1 family cathepsin B24, (**f**) chymotrypsin and (**g**) trypsin-like serine protease of larvae extracts as a function of intake type and feeding duration. Different letters indicate statistically significant differences at *p* = 0.05 (Tukey test).

Comparing the relative contents of α-amylase, it can be observed that feeding the larvae with wheat, sorghum and whole rice flour led to an overall reduction in enzyme levels with time. Figure 4 shows that after 5 h, 5 days and 10 days of feeding, the lowest amounts of α-amylase were recorded in larvae fed with rice flour, whereas larvae fed with sorghum flour *S35* (the sorghum cultivar with the high ATI content) exhibted higher relative contents of α-amylase. Comparing the relative content after 5 h of feeding, it appears that the larvae fed with wheat cultivar *Esperya* (with the high ATI content) presented significantly higher relative α-amylase contents compared to those fed with low-ATI wheat (*Siyazan* cultivar). It can be seen that the relative content of larvae α-amylase decreased drastically with time. Considering the group of larvae fed with both sorghum flours, a decrease of 50% of the relative amylase quantities was observed between 5 h and 5 days. During the same period, the decrease was even more pronounced for the larvae fed with rice flour, with the relative content of α-amylase being about 92% lower after 5 days compared to those registered at 5 h (Figure 4a).

The relative abundance of proteases yielded different results during the experiments. CLIP domain-containing serine protease, modular serine protease zymogen and C1 family cathepsin B24 showed similar profiles to amylase. In fact, a decrease in the relative abundance of these enzymes with time was observed, being particularly significant between the fifth hour and the fifth day of feeding, with decreases of 54%, 44% and 39% for CLIP domain-containing serine protease, modular serine protease zymogen and C1 family cathepsin B24, respectively (Figure 4b,c,e). From the fifth to the tenth day, this decrease persisted, but to a relatively limited degree. Only larvae fed with rice showed a slight increase in modular serine protease zymogen and C1 family cathepsin B24 after 10-day feeding, exhibiting a value of 233,472 ± 29,071 PA/mg larvae, compared to 178,143,6 ± 906 PA/mg larvae on the fifth day. However, an average increase in the relative content with time of dipeptidylpeptidase I and chymotrypsin was recorded in the larvae fed with the different cereals. For example, compared to the initial content, the average dipeptidylpeptidase I content in larvae increased relatively by 36%, 87% and 154% after 5 h, 5 days and 10 days of feeding, respectively, whereas with chymotrypsin, the increase was approximately 1%, 28% and 99% after 5 h, 5 days and 10 days of feeding, respectively (Figure 4d,f). However, there is an exception to the general trend exhibited by the larvae fed with sorghum meal containing high ATI contents (cultivar *S35*). The relative content of the two enzymes remained relatively constant in the larvae throughout the feeding time, reaching 117,731 ± 4236, 158,708 ± 12,996 and 184,883 ± 7494 PA/mg larvae; and 288,699 ± 8159, 389,896 ± 2891 and 401,827 ± 20,851 PA/mg larvae after 5 h, 5 days and 10 days of feeding for dipeptidylpeptidase I and chymotrypsin, respectively. Finally, the case of trypsin is quite particular. It can be seen that although the content at t = 0 was 761,297 ± 19,091 PA/mg larvae, averages of 805,101 ± 41,102; 733,587 ± 28,828 and 718,927 ± 33,355 PA/mg larvae were registered after 5 h, 5 and 10 days, respectively (Figure 4g). This result indicates that the feeding duration, type of cereal and ATI content had no significant effect (*p >* 0.05) on the relative content of trypsin in larvae.

## 4. Discussion

Regarding the trends of the average weight of the larvae during the feeding experiments, significant differences were observed only within the 5-day group. This result could be partially explained by the different levels of food intake. For the larvae fed with *Damougari* sorghum flour, a significantly lower food intake was observed, thus inducing a significantly lower weight in this group of larvae, compared to those fed with wheat flour. This was also observed when comparing the wheat-fed larvae with the rice-fed larvae. Feeding the larvae with wheat flour for five days resulted in significantly higher larval weight gains compared to those for rice flour. The fact that the protein content of the wheat samples was about two times higher could be responsible for the observed results. The death rate of larvae was also documented and differences between larvae were observed only after a period of 10 days. Although the larvae fed on wheat and sorghum flours with high ATI content (*Esperya* and *S35*) resulted in an increase in mortality compared to larvae fed on flours with low ATI content (*Siyazan* and *Damougari*), this difference was not always significant in all groups of larvae. The same was true for larvae fed with rice flour. The observed trends still suggest that cereal ATIs could have an influence on the mortality rate of larvae. However, it would also be interesting to consider the amount of flour being consumed. Wheat, sorghum and whole rice flours differ not only in terms of their protein content, but also in terms of their relative ATI content. It was previously shown that mortality and weight of *T. molitor* larvae was influenced by the level of proteins [42]. Franco et al. [43] established that ATIs led to an alteration of larval development; showing that ATI contents 0.19 and 0.53 lead to a significant reduction in larval survival. However, the results obtained in this work do not clearly establish a probable negative effect of ATI content on the weight gain of the larvae. Furthermore, feeding wheat meal to the larvae resulted in a shorter development time compared to sorghum meal, as indicated by an increased percentage of live pupae after 10 days (Figure 2c). The fact that the protein content was up to two times higher in wheat flour compared to sorghum could provide an explanation. This hypothesis is supported by a study by van Broekhoven et al. [42], in which higher protein content in the feed resulted in a shortening of larval development time.

Protein concentration was determined for both sodium phosphate buffer and ammonium bicarbonate/urea extracts. Ammonium bicarbonate/urea extracts yielded protein concentrations from 20 to 37 mg/mL, significantly higher than the values obtained from the sodium phosphate buffer extracts (8 to 9 mg/mL). A comparison of the two methods also reveals that protein concentrations differed between experimental groups. For example, significantly higher protein concentrations were obtained from extracts of larvae fed with low-ATI wheat flour (*Siyazan*) for a period of 10 days (7.90 ± 0.16 mg/mL), whereas the 5 h group exhibited values of 9.33 ± 0.04 mg/mL when using sodium phosphate buffer. Conversely, significantly lower protein concentrations were measured for the 10-day group (19.78 ± 0.16 mg/mL) compared to the 5-h group (27.05 ± 0.16 mg/mL) in the ammonium bicarbonate/urea extracts. Therefore, based on protein concentrations alone, it was difficult to draw concrete conclusions on the effect of meals’ ATI compositions on larval protein contents. However, the Kjeldahl method revealed total protein levels ranging from 43% to 53%, which are in agreement with those of Siemianowska et al. [4] and Morales-Ramos et al. [44], who also recorded total protein levels of 45% to 53%. The composition of the flours and their possible influence on the protein content of the larvae should also be considered. Although wheat flours contain about twice as much protein as sorghum and rice flours, it was observed that the total protein values of the larvae, as determined by the Kjeldahl method, fluctuated in a relatively narrow range during the different experimental periods. For this reason, the results suggest that the amount of protein contained in the meals did not have a direct effect on the protein content of the larvae. This hypothesis is also supported by a study by van Broekhoven et al. [42], who showed that the protein contents of *T. molitor* larvae fed with up to three times more protein in the meal did not differ significantly from each other. Behmer et al. [24] reported that insects have intrinsic regulatory mechanisms, allowing them to maintain their protein balance.

Analyzing the α-amylase activity, significant differences were observed in some cases within the experimental groups, although the values fluctuated overall in the range of 2.16 ± 0.06 to 3.27 ± 0.02 mM maltose/min (Figure 3a). Here, too, variation in grain ATI levels did not have a significant effect on larval enzymatic activity. However, the relative abundance of α-amylase, as determined by mass spectrometry, showed significant differences, especially between the experimental periods. Feeding the larvae with wheat, sorghum and rice flours resulted in a significant reduction of relative α-amylase content between the initial time (t = 0), the fifth hour and the fifth day. In contrast, the obtained amounts compared between the 5-day and the 10-day groups were more or less similar. This may potentially be explained by an adaptation mechanism of the larvae to the α-amylase inhibitors present in the meals. Silva et al. [35] showed that insects were able to accommodate the available feed, and the presence of α-amylase inhibitors resulted in an increase in the expression of two enzyme isoforms. The results obtained after 5-h feeding showed significantly higher α-amylase levels for larvae fed with the wheat cultivar *Esperya* (high ATI content) compared to those fed with the wheat cultivar *Siyazan* (low ATI content). This observation, in conjunction with the possible adaptive strategy of the insects, suggests that a high ATI content led to the induction of α-amylase within the first hours. However, it is necessary to qualify this hypothesis, since significantly higher levels of α-amylase were obtained from larvae fed with sorghum flour and after 5 and 10 days of feeding no more significant differences were reported.

The effect of wheat, sorghum and rice flours on larval protease levels showed significant decreases in CLIP domain-containing serine protease, modular serine protease zymogen and C1 family cathepsin B24 levels between the fifth hour and the fifth day of feeding (Figure 4b,c,e). These levels subsequently remained stable between days 5 and 10. For this reason, it can be assumed that the composition of the wheat, sorghum and whole rice flours also led to an inhibition of the activity or expression of these proteases in the larvae during the first hours of treatment, indicating the existence of larval defense strategies against the protease inhibitors. Such protease alteration could have a direct effect on the developmental characteristics of the larvae, knowing that the breakdown of dietary proteins provides the larvae with essential amino acids that are important for growth, but also for their development [45]. In contrast to the previous result, dipeptidylpeptidase I and chymotrypsin levels exhibited a gradual increase, whereas trypsin levels remained constant throughout the feeding duration (Figure 4d,f,g). These results are similar to those of Kuwar et al. [34], who showed that in the presence of a soybean trypsin inhibitor, an increase in chymotrypsin was observed in insects, whereas at the same time the trypsin level was reduced. An increase in protein-cleaving enzymes in the digestive tract and an increase in inhibitor-insensitive proteases are possible strategies by which insects could counteract this alteration. It is well known that ATIs are α-amylase/trypsin bi-functional inhibitors, and considering the composition of the different cereal flours used in this work in terms of their ATI content, it was difficult to find a clear trend showing a direct effect of these inhibitors on the expression of larval proteases.

## 5. Conclusions

In this study, the effect of five different cereal ATIs on the growth parameters and digestive enzyme contents of *T. molitor* larvae were investigated. The larvae were fed with wheat, sorghum and rice meals containing low and high levels of inhibitors. The feeding experiments were conducted over ten days, and the larvae developmental characteristics were documented. The results indicate that larvae fed with sorghum flour *Damougari* (with a lower ATI content) led to the lowest larval weight gains, whereas those fed with higher-ATI-content wheat meal exhibited higher weight gains. Similarly, feeding the larvae with sorghum meal resulted in a significant reduction of the pupation compared to wheat and rice flours. We observed a significant increase in the larval death rate after 10 days of feeding with all meal samples. Taking into account the protein content, it emerged that the patterns of the specific activities according to the type of feed provided to the larvae were similar to those of the amylase activities. The developed HPLC-MS/MS method allowed the identification of larval digestive enzyme biomarkers and thus their relative quantification. Feeding the larvae with wheat, sorghum and rice meals resulted in a significant reduction in the relative content of α-amylase, CLIP domain-containing serine protease, modular serine protease zymogen and C1 family cathepsin B24 between the initial time and the fifth day, whereas the amounts of dipeptidylpeptidase I and chymotrypsin increased. The content of trypsin remained constant during the entire feeding period. Since no correlation between the ATI content of the analyzed flour samples and the relative content of the digestive enzymes of the larvae could be clearly established, we could not conclusively determine to what extent the cereal ATI content contributed to the variations in the relative larval α-amylase and proteases contents. Therefore, it cannot be excluded that other ingredients also present in wheat, sorghum and whole rice flours have some effect on the regulation of digestive enzymes and thus influence the growth of larvae. All these results indicate that the rearing of larvae may be influenced by the feeding strategy, and considering the results, wheat can be recommended for feeding. Feeding strategies in turn may provide a tool for changing the nutritional quality of meal worms when considering them as an alternative protein source for food and feed purposes.

## Figures and Tables

**Table 1:** Protein content and relative ATI content of selected feed samples.

Sample	Cultivar	Protein Content * (g/100 g)	ATI Content **	
			(PA/mg Flour)	(PA/µg Protein)
Wheat	*Siyazan*	15.6	79,431 ± 1218	931 ± 14
	*Esperya*	13.9	1,460,159 ± 95,183	25,231 ± 1645
Sorghum	*Damougari*	8.5	14,903 ± 223	935 ± 14
	*S35*	7.9	147,671 ± 2509	11,017 ± 187
Rice	n.a.	7.9	473,317 ± 5349	17,174 ± 193

* total protein content of samples was evaluated according to the Kjeldahl method (N × 6.25); ** ATI amounts are given in terms of relative content, as described in our previous works [30,39]. Tables S1–S3 (Supplementary Data) present the optimized conditions of the HPLC-MS/MS method used for the quantification of wheat, sorghum and rice ATIs. n.a.: not available.

**Table 2:** Protein content from sodium phosphate and Ambi/urea extracts, and total protein content, determined according to the Kjeldahl method, of larvae after 5 h, 5 days and 10 days (n = 3).

				Feeding Samples				
			I.L.	Rice	*Siyazan*	*Esperya*	*Damougari*	*S35*
Protein content (mg/mL)	Na_3_PO_4_ buffer	t = 0	9.42 ± 0.19	-	-	-	-	-
		5 Hours	-	7.80 ± 0.09 ^a^	7.90 ± 0.16 ^a,b^	8.22 ± 0.04 ^b^	8.20 ± 0.22 ^a,b^	8.80 ± 0.10 ^c^
		5 Days	-	7.94 ± 0.18 ^a^	8.97 ± 0.28 ^b^	8.82 ± 0.25 ^b^	8.40 ± 0.23 ^c^	8.05 ± 0.19 ^a,c^
		10 Days	-	8.50 ± 0.08 ^a^	9.33 ± 0.04 ^b^	9.02 ± 0.08 ^b^	8.06 ± 0.07 ^c^	8.49 ± 0.30 ^a^
	Ambi/urea buffer	t = 0	34.93 ± 0.44					
		5 Hours	-	26.73 ± 0.72 ^a,b^	27.05 ± 0.16 ^b^	25.21 ± 0.69 ^a^	29.45 ± 0.11 ^c^	26.56 ± 0.71 ^a,b^
		5 Days	-	37.47 ± 1.38 ^a^	32.74 ± 0.21 ^b^	26.91 ± 1.01 ^c^	34.75 ± 0.38 ^d^	33.97 ± 0.75 ^b,d^
		10 Days	-	22.39 ± 0.74 ^a^	19.78 ± 0.16 ^b^	19.95 ± 0.34 ^b,c^	30.41 ± 0.21 ^d^	27.48 ± 0.75 ^e^
Total Protein (g/100 g)		t = 0	53.4	-	-	-	-	-
		5 Hours	-	48.6	49.8	49.7	49.9	48.7
		5 Days	-	45.1	46.0	46.8	48.1	44.8
		10 Days	-	44.0	45.7	47.5	45.6	43.9

I.L.: initial larvae before starting with the feeding experiments. t = 0 represents the initial larvae at the starting conditions. Rows within the group labeled with a different letter were significantly different at *p* = 0.05 (Tukey test).

## Supplementary Materials

The following are available online at https://www.mdpi.com/article/10.3390/insects12050454/s1. Figure S1: Behavior of larvae fed with rice whole meal, wheat flours *Siyazan* and *Esperya*, and sorghum flours *Damougari* and *S35* during the feeding experiments. Figure S2: Time sequence of the feeding experiment. Figure S3: Linearity of the HPLC-MS/MS measurements of (a) α-amylase, (b) trypsin-like serine protease, (c) C1 family cathepsin B24, (d) dipeptidylpeptidase I, (e) chymotrypsin 1, (f) CLIP domain-containing serine protease and (g) modular serine protease zymogen. Table S1: The optimized conditions for the multiple reaction monitoring (MRM) for the final HPLC-MS/MS method. Table S2: The optimized conditions of the HPLC-MS/MS method for the analysis and relative quantification of ATIs in sorghum samples. Table S3: The optimized parameters of the applied MRM method for the analyzed rice ATIs. Table S4: Characteristics of different enzymes analyzed from *Tenebrio molitor* larvae (yellow mealworm beetle). Table S5 Final optimal parameters used to quantify the relative abundance of mealworms α-amylase and proteases by the targeted LC-MS/MS method.
