# Supplementary material for: Effect of Cereal α-Amylase/Trypsin Inhibitors on Developmental Characteristics and Abundance of Digestive Enzymes of Mealworm Larvae (Tenebrio molitor L.)
Source: Insects. 2021 May 14;12(5):454. doi: 10.3390/insects12050454 (PMC8156395; doi:10.3390/insects12050454)
Supplement: Supplementary file 1 [file insects-12-00454-s001.zip › insects-1217780-supplementary.pdf]

Supplementary Material

Effect of cereal α-amylase/trypsin inhibitors on developmental characteristics and abundance of digestive enzymes of meal-worm larvae (*Tenebrio molitor* L.)

Sorel Tchewonpi Sagu[1], Eva Landgräber[1], Ina M. Henkel[2], Gerd Huschek[3], Thomas Homann[1], Sara Bußler[4], Oliver K. Schlüter[4,5] and Harshadrai Rawel[1,*]

[1]Institute of Nutritional Science, University of Potsdam, Arthur-Scheunert-Allee 114-116, 14558 Nuthetal, Germany; sorelsagu@uni-potsdam.de (S.T.S.); e.landgraeber@live.de (E.L.), homann@uni-potsdam.de (T.H.)

[2]EntoNative GmbH, Arthur-Scheunert-Allee 40-41, 14558 Nuthetal, Germany; ina.henkel@tenetrio.de (I.M.H.)

[3]IGV-Institut für Getreideverarbeitung GmbH, Arthur-Scheunert-Allee 40/41, Nuthetal OT, D-14558 Bergholz-Rehbrücke, Germany; gerd.huschek@igv-gmbh.de (G.H.)

[4]Leibniz Institute for Agricultural Engineering and Bioeconomy (ATB), Quality and Safety of Food and Feed, Max-Eyth-Allee 100, 14469 Potsdam, Germany; sbussler@gnt-group.com (S:B.); oschlueter@atb-potsdam.de (O.K.S.)

[5]University of Bologna, Department of Agricultural and Food Sciences, p.zza Goidanich 60, 47521, Cesena, Italy; oliver.schluter@unibo.it (O.K.S.)

* Corresponding author.

Tel: +49-33200-88-5525/5578 (H.R.)

E-mail: rawel@uni-potsdam.de

Content

Figure S1 Behaviour of larvae in rice whole meals (blue boxes); wheat flours Siyazan and Esperya (green boxes); sorghum flours Damougari and S35 (white boxes) during the feeding experiments

Figure S2 Time sequence of the feeding experiment.

Figure S3. Linearity of the HPLC-MS/MS measurements of (a) α-amylase, (b) trypsin-like serine protease, (c) C1 family cathepsin B24, (d) dipeptidylpeptidase I, (e) chymotrypsin 1, (f) CLIP domain-containing serine protease and (g) modular serine protease zymogen.

Table S1 The optimized conditions for the multiple reaction monitoring (MRM) for the final HPLC-MS/MS method

Table S2 The optimized conditions of the HPLC-MS/MS method for the analysis and relative quantification of ATIs in sorghum samples.

Table S3 The optimized parameters of the applied MRM method for the analyzed rice ATIs.

Table S4 Characteristics of different enzymes analyzed from *Tenebrio molitor* larvae (Yellow mealworm beetle)

Table S5 Final optimal parameters used to quantify the relative abundance of mealworms α-amylase and proteases by the targeted LC-MS/MS method

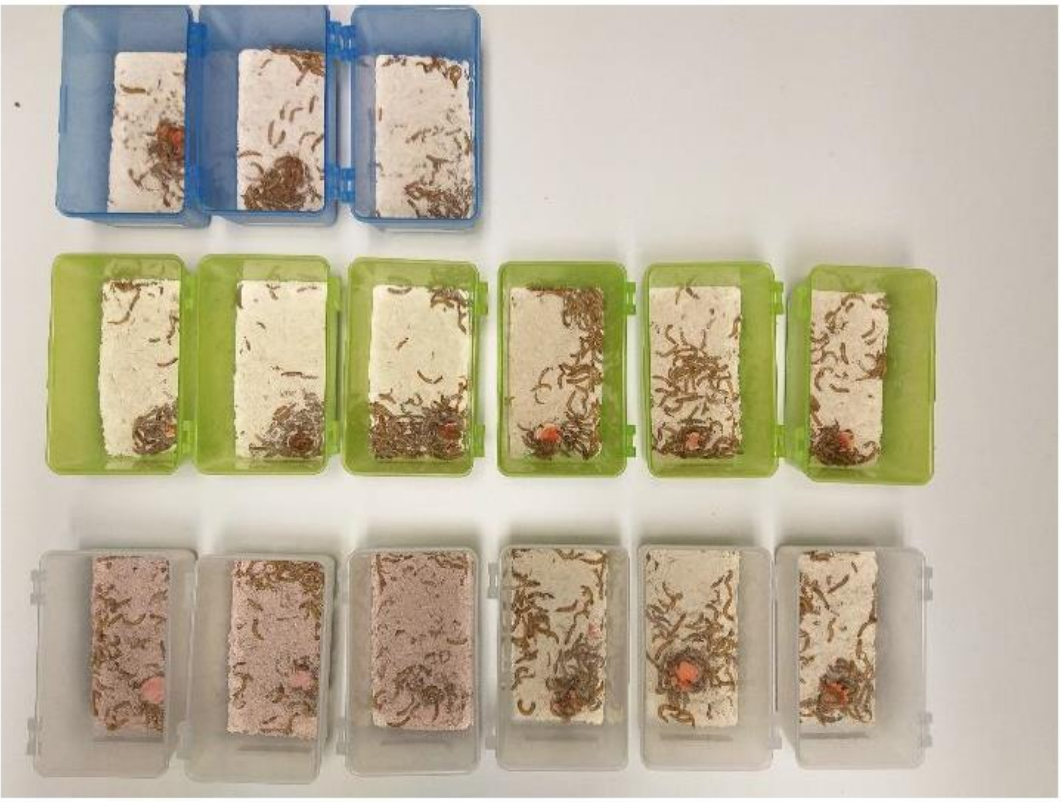

Figure S1 Behaviour of larvae in rice whole meals (blue boxes); wheat flours *Siyazan* and *Esperya* (green boxes); sorghum flours *Damougari* and *S35* (white boxes) during the feeding experiments

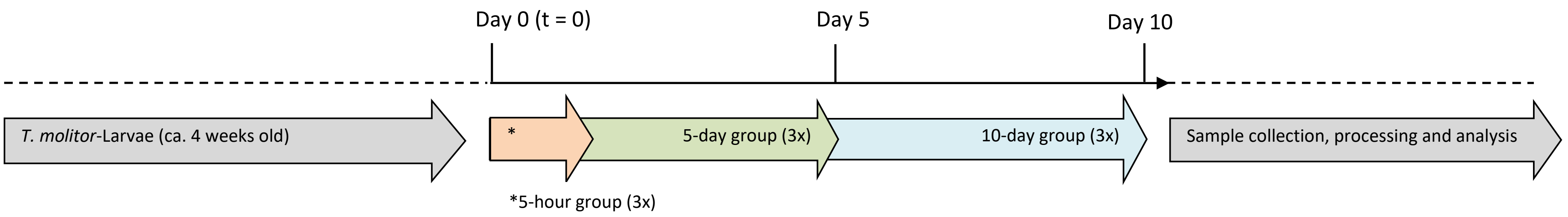

<u>Conditions before starting the experiment</u>

- Feed to *Tenebrio molitor* larvae ratio: 2:1
- Basic feed: wheat
- Room temperature: 25 °C
- Relative humidity: 50-60 %

<u>Starting the experiment</u>

- Weighing flour: 20 g
- Weighing larvae: 10 g

<u>End of experiment</u>

- Documentation of the developmental characteristics
- Collect and processing the larvae for the further biochemical analysis and relative quantification of larvae digestive enzymes.

Figure S2 Time sequence of the feeding experiment.

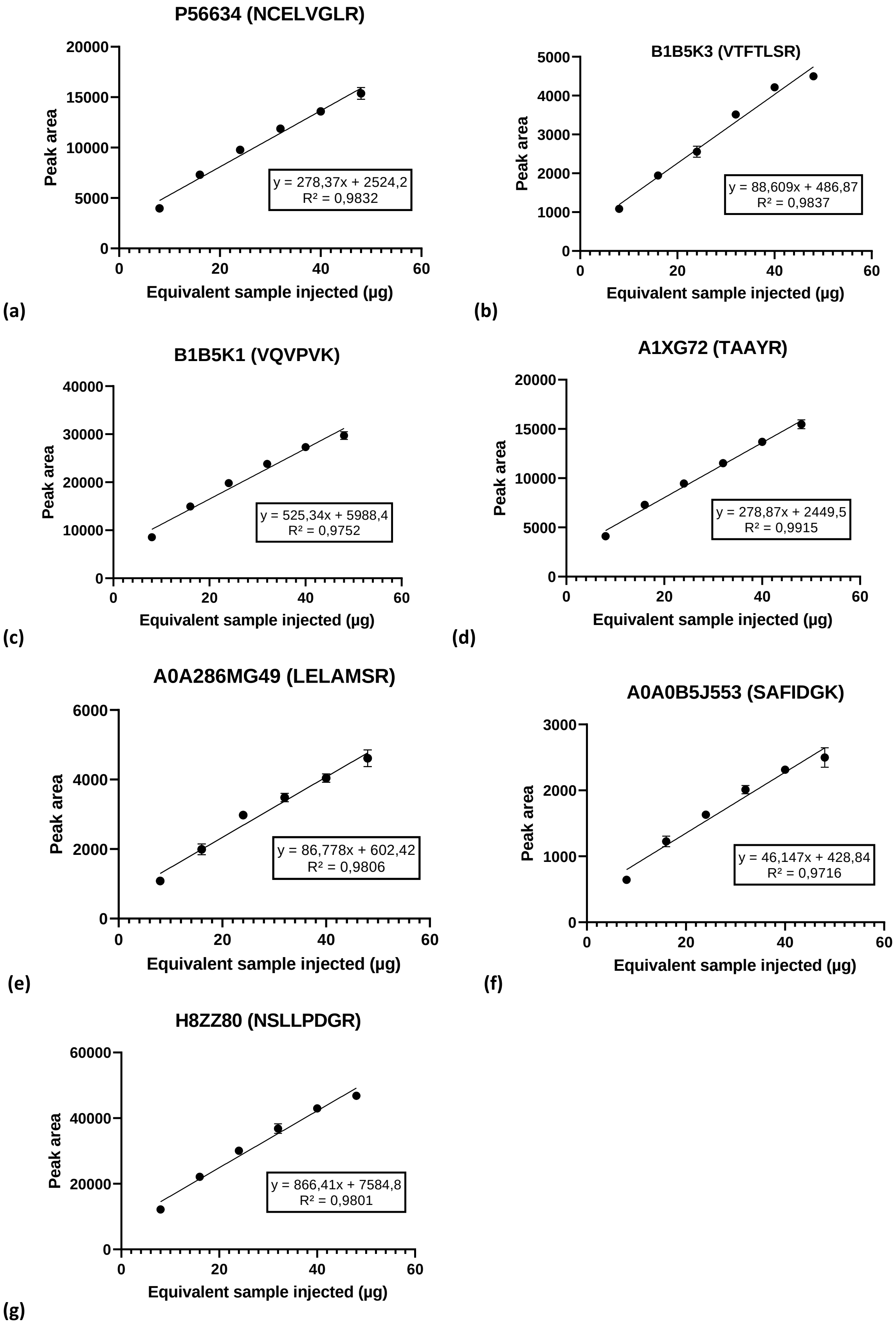

Figure S3. Linearity of the HPLC-MS/MS measurements of (a) α-amylase, (b) trypsin-like serine protease, (c) C1 family cathepsin B24, (d) dipeptidylpeptidase I, (e) chymotrypsin 1, (f) CLIP domain-containing serine protease and (g) modular serine protease zymogen.

Table S1 The optimized conditions of the multiple reaction monitoring (MRM) for the analysis of relative abundance of wheat ATIs by HPLC-MS/MS method

Protein	Biomarkers	Fragment	Q1-Mass	Q3-Mass	CE [eV]	Retention time [min]
**P01083**	K.VSALTGCR.A	A[y6]	432.2^{++}	677.3^{+}	14.4	8.0
		L[y5]		606.3^{+}	14.4	
		T[y4]		493.2^{+}	14.4	
**P01083**	K.VPIPNPSGDR.A	P[y9]	526.3^{++}	952.5^{+}	23.3	9.1
		P[y7]		742.3^{+}	23.3	
		P[y5]		531.3^{+}	26.3	
**P17314**	R.TNLLPHCR.D	L[y6]	505.8^{++}	795.4^{+}	19.7	8.6
		P[y4]		569.3^{+}	19.7	
		L[b3]		329.2^{+}	19.7	
**P16850**	R.SDPNSSVLK.D	P[y7]	473.7^{++}	744.4^{+}	15.7	8.1
		S[y5]		533.3^{+}	21.7	
**P01084/85**	K.LTAASITAVCR.L	T[y10]	581.8^{++}	1049.5^{+}	25.0	10.2
		S[y9]		806.4^{+}	22.0	
		T[y5]		606.3^{+}	25.0	
**P15851**	R.TSDPNSGVLK.D	P[y7]	509.3^{++}	714.4^{+}	19.8	8.1
		S[y5]		503.3^{+}	25.8	
		P[y7]		357.7^{++}	19.8	
**P16159**	R.YFMGPK.S	F[y5]	371.7^{++}	579.3^{+}	12.5	9.7
		M[y4]		432.2^{+}	12.5	
		G[y3]		301.2^{+}	12.5	
		P[y2]		244.2^{+}	12.5	
**P16159**	R.EVQMDFVR.I	Q[y6]	512.3^{++}	795.4^{+}	16.9	10.9
		M[y5]		667.3^{+}	16.9	
		D[y4]		536.3^{+}	13.9	
		F[y3]		421.3^{+}	25.9	
**P81496-Q43723-Q43691**	R.EQCVPGR.E	C[y5]	423.2^{++}	588.3^{+}	14.0	6.85
		V[y4]		428.3^{+}	14.0	
		P[y3]		329.2^{+}	14.0	
**P93602**	R.CEALR.V	E[y4]	324.7^{++}	488.3^{+}	14.1	6.95
		A[y3]		359.2^{+}	11.1	
		L[b4]		474.2^{+}	11.1	
**P83207**	R.ELAAISSNCR.C	A[y7]	560.8^{++}	807.4^{+}	18.4	8.9
		I[y6]		736.3^{+}	21.4	
		S[y5]		623.3^{+}	21.4	
		S[y4]		536.2^{+}	18.4	
**Q4U199**	K.LTAASITAVCK.L	T[y10]	567.8^{++}	1021.5^{+}	25.0	10.0
		S[y7]		778.4^{+}	22.0	
		T[y5]		578.3	25.0	
**Q41540**	R.NYVEEQACR.I	Y[y8]	608.3^{++}	1054.5^{+}	25.1	8.0
		V[y7]		891.4^{+}	19.1	
		E[y6]		792.3^{+}	19.1	
		E[y5]		663.3^{+}	19.1	
**Q41540**	R.IEMPGPPYLAK.Q	M[y9]	608.3^{++}	973.5^{+}	13.9	11.8
		P[y8]		842.5^{+}	28.9	
		G[y6]		745.4^{+}	19.9	
		P[y6]		688.4^{+}	13.9	

Q1 = Precursor mass; Q3 = Transition mass; CE = Collision energy

Table S2 The optimized conditions of the multiple reaction monitoring (MRM) for the analysis of relative abundance of sorghum ATIs by HPLC-MS/MS method.

Protein	Biomarkers	Q1-Mass	Fragment	Q3-Mass	CE [eV]	Retention time (min)
**P81367**	R.ELAAVPSR.C	421.7^{++}	A [y6]	600.3^{+}	14.1	8.6
			A [y5]	529.3^{+}	17.1	
			V [y4]	458.3^{+}	11.1	
			P [y3]	359.2^{+}	11.1	
**P81368**	R.TYMVR.R	335.2^{++}	Y [y4]	568.3^{+}	14.4	8.2
			M [y3]	405.2^{+}	8.4	
			Y [b2]	265.1^{+}	6.4	
**IS**	GWGG	376.1^{++}	W [y3]	319.1^{+}	12.7	8.7
			G [y2]	133.0^{+}	21.7	
			G [y1]	76.0^{+}	21.7	
			W [b2]	244.1^{+}	12.7	
			G [b3]	301.1^{+}	6.7	

Q1 = Precursor mass; Q3 = Transition mass; CE = Collision energy

Table S3 The optimized conditions of the multiple reaction monitoring (MRM) for the analysis of relative abundance of rice ATIs by HPLC-MS/MS method.

Protein	Biomarkers	Q1-Mass	Fragment	Q3-Mass	CE [eV]	Retention time (min)
**P29421**	R.VSTDVR.I	338.7^{++}	S [y5]	577.3^{+}	11.5	6.7
			T [y4]	490.3^{+}	8.5	
			D [y3]	389.2^{+}	11.5	
**Q8H4L8**	R.CDALNHMLR.I	565.3^{++}	D [y8]	854.5^{+}	21.5	9.8
			A [y7]	783.4^{+}	27.5	
			L [y6]	670.3^{+}	24.5	
**Q7X8H9**	R.WCAVAGGR.L	438.7^{++}	C [y7]	690.3^{+}	20.6	8.8
			A [y6]	530.3^{+}	14.6	
			V [y5]	459.3^{+}	20.6	
**Q7X7E6**	R.WCAAAGGR.M	424.7^{++}	C [y7]	662.3^{+}	20.2	7.9
			A [y6]	502.3^{+}	17.2	
			A [y5]	431.2^{+}	20.2	
**Q01883**	R.QLAAVDDGWCR.C	645.8^{++}	L [y10]	1049.4^{+}	27.0	10.6
			A [y9]	978.4^{+}	24.0	
			A [y8]	907.4^{+}	24.0	
**Q01882**	K.DQVVYSLGER.C	583.3^{++}	Q [y9]	1050.6^{+}	22.0	11.3
			V [y8]	922.5^{+}	22.0	
			V [y7]	823.4^{+}	22.0	
**Q01881**	R.GTAAAAEQVR.R	487.3^{++}	A [y8]	744.4^{+}	22.1	7.2
			A [y7]	673.4^{+}	19.1	
			A [y6]	602.3^{+}	13.1	
**Q2QLS3**	R.GPSCAVDITAR.C	573.8^{++}	P [y10]	1089.5^{+}	18.8	12.0
			S [y9]	992.5^{+}	15.8	
			C [y8]	905.5^{+}	21.8	
**Q8GVI3**	R.LLEDLPGCPR.E	585.3^{++}	L [y9]	943.4^{+}	22.1	10.8
			E [y8]	814.4^{+}	19.1	
			D [y7]	699.4^{+}	28.1	

Q1 = Precursor mass; Q3 = Transition mass; CE = Collision energy

Table S4 Characteristics of different enzymes analyzed from *Tenebrio molitor* larvae (Yellow mealworm)

Enzyme	Accession number	MW (kDa)	Length (a.a.)	Function
Alpha-amylase	P56634	51.240	471	Alpha-amylase activity
Trypsin-like serine protease	H8ZZ80	26.098	258	Serine-type endopeptidase activity
C1 family cathepsin B24	A0A0B5J553	35.335	320	Cysteine-type endopeptidase activity
Dipeptidylpeptidase I	A0A286MG49	44.506	403	Cysteine-type peptidase activity
Chymotrypsin 1	A1XG72	27.617	275	Serine-type endopeptidase activity
CLIP domain-containing serine protease	B1B5K1	41.910	383	Serine-type endopeptidase activity
Modular serine protease zymogen	B1B5K3	70.085	632	Serine-type endopeptidase activity

Table S5 Final optimal parameters used to quantify the relative abundance of mealworms α-amylase and proteases by the targeted LC-MS/MS method

Enzyme	Biomarkers	Fragment	Q1-Mass	Q3-Mass	CE [eV]	Retention time [min]
**P56634**	R.NCELVGLR.D	E [y6]	480.75^{++}	686.4^{+}	15.9	10.3
		L [y5]		557.3^{+}	21.9	
		V [y4]		444.2^{+}	15.9	
**B1B5K3**	R.VTFTLSR.R	F [y5]	412.2^{++}	623.2^{+}	13.8	12.2
		T [y4]		476.2^{+}	13.8	
		T [b2]		201.1^{+}	13.8	
**B1B5K1**	K.VQVPVK.Q	V [y4]	335.2^{++}	442.3^{+}	8.4	9.2
		P [y3]		343.2^{+}	8.4	
		Q [b2]		228.1^{+}	8.4	
**A1XG72**	R.TAAYR.S	Y [y2]	291.1^{++}	338.1^{+}	16.0	7.3
		R [y1]		175.1^{+}	13.0	
**A0A286MG49**	R.LELAMSR.R	L [y5]	410.2^{++}	577.3^{+}	13.7	6.8
		M [y3]		393.2^{+}	13.7	
**A0A0B5J553**	K.SAFIDGK.T	F [y5]	369.2^{++}	579.3^{+}	15.4	8.4
		D [y3]		319.1^{+}	12.4	
		G [y2]		204.1^{+}	9.4	
**H8ZZ80**	K.NSLLPDGR.I	G [y2]	436.2^{++}	232.1^{+}	23.5	7.9
		S [b2]		202.1^{+}	11.5	
		D [b6]		640.3^{+}	11.5	

Q1 = Precursor mass; Q3 = Transition mass; CE = Collision energy
